# Traumatic Fracture: Dislocation of Cervicothoracic Junction—Grand Round Presentation of C7-T1 Instabilities and Different Instrumentation Techniques

**DOI:** 10.1155/2020/7578628

**Published:** 2020-07-01

**Authors:** Mohammad A. Alsofyani, Soufiane Ghailane, Sultan Alsalmi, Sreenath Jakinapally, Louis Boissière, Ibrahim Obeid, Jean-Marc Vital

**Affiliations:** ^1^Department of Spinal Surgery Unit, Bordeaux University, Bordeaux University Hospital, C.H.U Tripode Pellegrin, Place Amélie Raba Léon, 33076 Bordeaux, France; ^2^Orthopedic Department, College of Medicine and University Hospital, University of Hail, P.O. Box 2440, Hail City, Saudi Arabia; ^3^Department of Neurosurgery, Amiens University Medical Center, Amiens University, Amiens, France; ^4^Department of Neurosurgery, Imam Abdulrahman Bin Faisal University, Dammam City, Saudi Arabia

## Abstract

**Introduction:**

Acute traumatic cervicothoracic junction spinal lesions are rare disorders and poorly documented. We report a case of a traumatic cervicothoracic fracture-dislocation. We present our experience in the operative treatment of an unstable fracture-dislocation at the cervicothoracic junction. *Materials and Method*. A seventy-year-old man was transferred to our hospital. We found paresthesia in the corresponding dermatome of C7 and C8 bilaterally. Initial CT scan shows vertebral body fracture of T1 with retropulsion into the spinal canal and anteroposterior dislocation of cervicothoracic junction type C according to AOSpine subaxial injury. Traumatic disc material at C7-T1 was removed by anterior cervical discectomy and fusion of C6-T2. Fixation was done from C6 to T2 in the prone position.

**Results:**

At one-year postoperative follow-up, radiographs revealed bony fusion at the level of C7-T1, and the patient had no major functional disability.

**Conclusion:**

We opted for the ventral-dorsal approach in our case for maximum stabilization and to prevent mechanical complications.

## 1. Introduction

Traumatic injuries at the cervicothoracic junction (CTJ) are a relatively rare event and considered as a significant cause of paraparesis or paraplegia posttraumatic. As young people are most commonly injured, it is considered as a significant economic burden to the family and society. In CTJ injuries, surgical techniques and associated complications have been extensively described in the literature during the past decade, whereas the choice of anterior versus posterior or double-stage fixation has been given little attention [1–3]. The purpose of this study is to discuss different instrumentation techniques of this rare injury.

## 2. Case Presentation

A seventy-year-old male was transferred to our hospital after sustaining trauma falling down into a watercourse. The initial Glasgow Coma Scale score was 15. Upon examination, he was hemodynamically stable and breathing spontaneously; we found paresthesia corresponding with dermatomes C7 and C8 bilaterally. The rest of the neurological physical examination was normal, and the patient was categorized grade E based on the American Spinal Injury Association classification.

**Figure 1:**
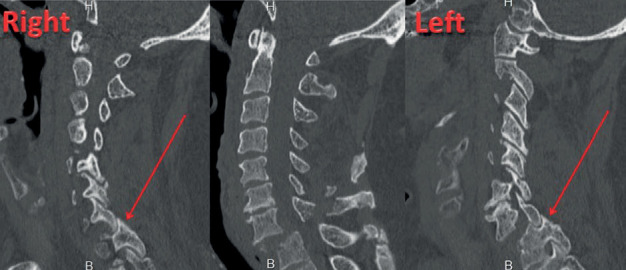
Preoperative CT scan of the patient shows vertebral body fracture of T1 and biarticular dislocation of the cervicothoracic junction.

Cervical spine radiography and two-dimensional reconstructed computed tomography (CT) scans (Figure 1) showed vertebral body fracture of T1 and anteroposterior dislocation of CTJ type C according to AOSpine subaxial injury.

Under general anesthesia, an image intensifier was used, and 16 kilograms of halo traction was applied with full muscle relaxation; the dislocation was irreducible. Iliac crest autogenous bone grafting was harvested. A left long presternocleidomastoid to the midline approach was then made for exposing the T2 vertebral body. Traumatic disc material at C7-T1 was removed by anterior cervical discectomy. Then, as the patient had biarticular dislocation, we placed the Caspar distractor on the medial line. The dislocation was reduced partially by increasing gently the distraction. When the facets were point to point on the oblique fluoroscopic view, we pushed gently on the upper vertebra, and the reduction was achieved on the oblique fluoroscopic. Anterior fusion from C6 to T2 was performed by iliac crest bone graft and anterior plate. This technique of reduction is successful in 34% of uniarticular dislocations and 27% of biarticular dislocations [4].

**Figure 2:**
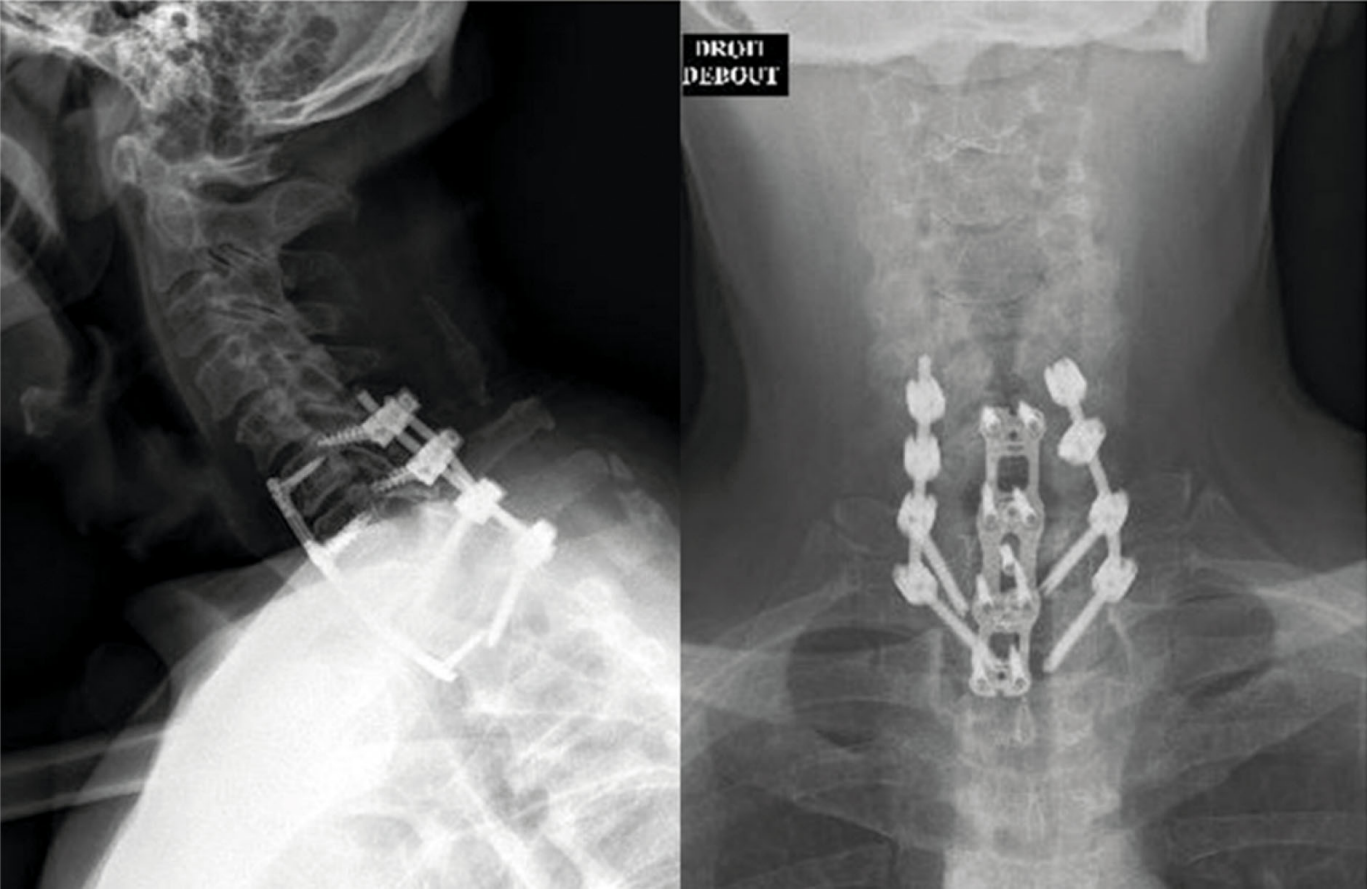
The patient underwent a C6-T2 fixation, followed by posterior C6-T2 arthrodesis.

**Figure 3:**
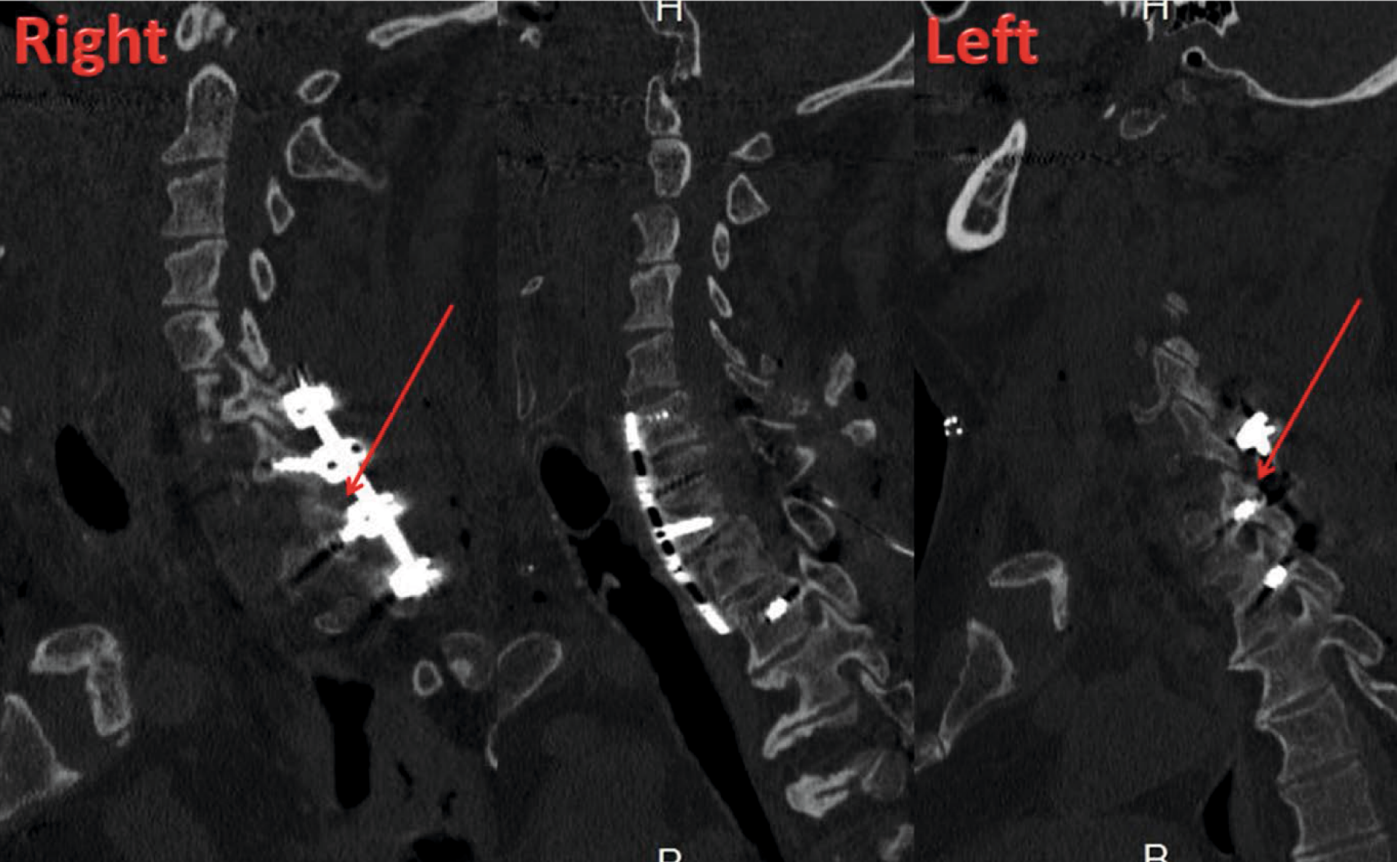
Postoperative CT scan shows complete reduction of cervicothoracic dislocation.

**Figure 4:**
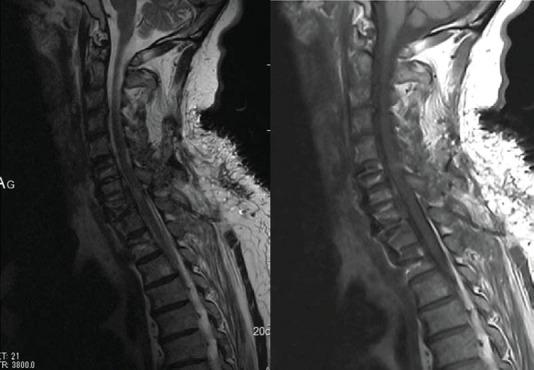
MRI postoperative does not show any abnormality of the spine canal.

Secondly, for increasing the stability of CTJ, the patient was turned to the ventral position. Fixation was done without facetectomy associated with instrumentation from C6 to T2. Screws were placed in the lateral mass of C6 and pedicular screws from C7 to T2. Cervical spine radiography (Figure 2) and computed tomography (CT) scans (Figure 3) postoperative showed complete reduction of CTJ. In the immediate postoperative period, the patient complained of transient paraplegia; postoperative magnetic resonance imaging (MRI) (Figure 4) did not show mechanical compression or epidural hematoma. The transient paraplegia disappeared on the first postoperative day. The patient was discharged to the rehabilitation center with an immobilisation collar for 2 months.

**Figure 5:**
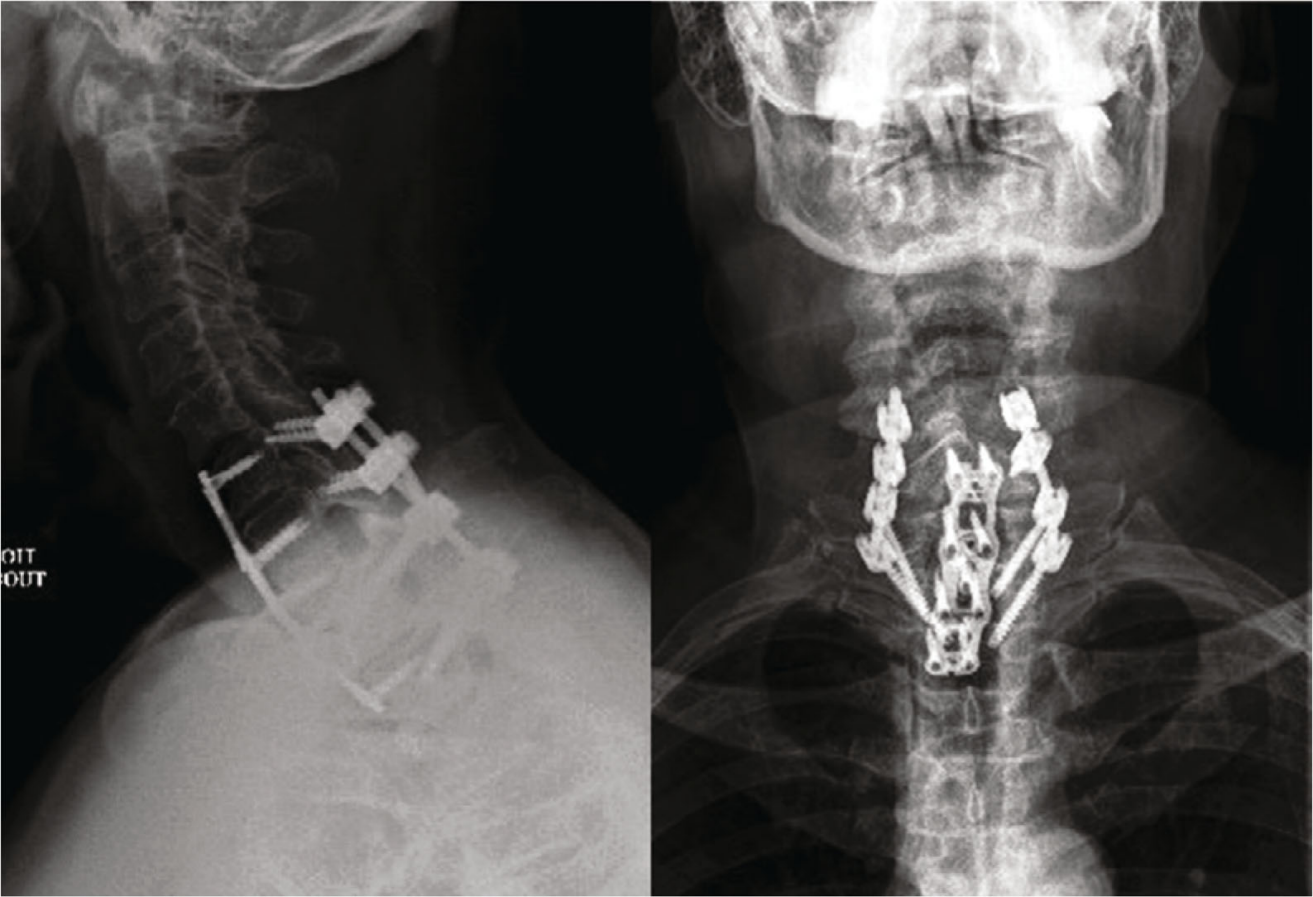
X-ray at one-year follow-up shows a well bony fusion.

At one-year follow-up, cervical spine radiography (Figure 5) revealed a well-reduced CTJ with bone graft healing; the patient had no major functional trouble.

## 3. Discussion

CTJ is a region in the vertebral column where biomechanically, it represents a region where there is an inflection from mobile cervical lordosis to rigid thoracic kyphosis. Radiographically, it is not easy to visualize especially in traumatic cases. Traumatic injuries to the CTJ usually include fracture-dislocation or isolated fracture [5–7]. Immediate treatment includes closed reduction, followed by surgical fixation [6, 8]. The different anatomical features of CTJ [3, 9], with the peculiar biomechanical characteristics [10], require the need of a suitable surgical technique for maximum stabilization.

Nichols et al. [11] reported a 9% incidence of CTJ injury, described as unilateral and bilateral facet dislocations or true fracture-subluxation of C7-T1. Evans [12] in 1983 published one of the largest reviews to date, where he investigated 14 cases determined from 587 cervical spinal cord injuries (SCIs) during a 26-year period, providing an incidence of 2.4% of cervical SCIs.

Dislocations of the CTJ are frequently missed especially in simple cervical trauma cases visiting the emergency department. Evans [12] confirms that almost two-thirds were not well diagnosed on admission. Similar arguments have already been focused and discussed in a series of three-hundred cervical spine traumas by Bohlman [13], published over forty years ago. Traumatic lesions at the CTJ are easily missed, due to poor visualization in this area; other imaging studies such as swimmer's view, CT reconstruction, or MRI should be carried out in suspicious cases [7].

Following trauma at CTJ, neurological problems are commonly seen. This may be due to the anatomical features of the upper thoracic spine through the small canal size, even though vascular insufficiency through the low blood supply of the lower cervical spine makes it more susceptible to ischemic injury.

A large and growing body of literature has investigated the successful results for anterior instrumentation for pathological fractures of the CTJ; the reported results of anterior instrumentation for traumatic fractures and dislocations are limited [14]. More recent attention has focused on biomechanical features regarding the adequacy of anterior instrumentation for stabilizing posterior element injuries of the cervical spine [15, 16]. Bueff et al. [17] reported in a cadaveric study that anterior fixation techniques had less stiffness than posterior fixation, especially in resisting flexion-distraction forces. Boockvar et al. [2] demonstrated that anterior instrumentation alone may not be enough biomechanically for CTJ and that combined anterior-posterior fixation and fusion may be considered to increase stabilization for fusion success.

Anterior approaches can be practically classified into a low anterolateral cervical approach, a transmanubrial-transsternal approach, and a thoracotomy [14]. The way of choosing the approach depends on the level of lesion and the surgeon's experience. The low cervical approach is widening of the classical cervical approach medial to sternocleidomastoid (SCM); this allows access to the first and the second thoracic vertebral body [14, 18]. The transmanubrial-transsternal approach is required for the lesion down to T3 [14]. It can be done with low cervical approach for accessing the lower cervical spine. Several modifications were done for decreasing the high risk of morbidity by this approach [14]. Pointillart et al. [19] modified the approach by extending the standard anterolateral cervical approach caudally. Preoperative planning of MRI sagittal slices is mandatory as described by Sharan et al. [20]. If the sternum restricts the access, a median manubriotomy gives adequate access to the spine. The advantages of this modified approach are to protect the clavicles, sternoclavicular joints, and sternal body. If the lesion is below the T4 level, a thoracotomy has to be performed.

Posterior approach to the CTJ has been performed largely because of the technical difficulty of the anterior approach. Practically posterior instrumentation of the CTJ should always be fused, because of the instability of the CTJ. In the cervical spine, lateral mass screws are often performed in the posterior fixations. The thickness of the lateral mass is decreased from C5 to C7 from 11 to 8.7 mm [21]. Inversely, the size of the pedicle increases progressively from the low cervical spine to the thoracic spine. The pedicle width increases from C5 to C7 from 5.2 mm to 6.5 mm [21]. The angle decreases from 4 to 6 in each level from C5 to T1 between the vertebral body and the axis of the pedicle, decreasing from 50 at the C5 to 34 at the T1 [21]. Instrumentation of pedicle screws must estimate these differences in angulation. Posterior instrumentation can bring back most of the stability in a two-column but not a three-column injury [22].

Collectively, in our case, we started with the anterior approach to reduce the dislocation from the front as it is less invasive comparing with posterior facetectomy and we have direct exposure of the body and disc. Then, we have instrumented the cervical spine posteriorly to decrease the risk of instability and mechanical complications as the patient had a three-column injury of CTJ. Our case report with the grand round presentation of the literature outlines the importance of anterior versus posterior or double-stage fixation at the CTJ. Our case presented with a three-column injury and has shown the importance of previous literature for understanding the stability at this unique region [15–17, 22].
